# Hydrogen Concentration Distribution in 2.25Cr-1Mo-0.25V Steel under the Electrochemical Hydrogen Charging and Its Influence on the Mechanical Properties

**DOI:** 10.3390/ma13102263

**Published:** 2020-05-14

**Authors:** Changdong Yin, Jianjun Chen, Dongdong Ye, Zhou Xu, Jiahao Ge, Haiting Zhou

**Affiliations:** 1 School of Mechanical and Power Engineering, East China University of Science and Technology, Shanghai 200237, China; y30180365@mail.ecust.edu.cn (C.Y.); Y10170088@mail.ecust.edu.cn (D.Y.); y45180021@mail.ecust.edu.cn (Z.X.); Y45170004@mail.ecust.edu.cn (J.G.); 2 Department of Quality and Safety Engineering, China Jiliang University, Hangzhou 310018, China; zhouhaiting@cjlu.edu.cn

**Keywords:** electrochemical hydrogen permeation, galvanostatic hydrogen charging, diffusible hydrogen content, mechanical properties evaluation, ABAQUS simulation

## Abstract

The deterioration of the mechanical properties of metal induced by hydrogen absorption threatens the safety of the equipment serviced in hydrogen environments. In this study, the hydrogen concentration distribution in 2.25Cr-1Mo-0.25V steel after hydrogen charging was analyzed following the hydrogen permeation and diffusion model. The diffusible hydrogen content in the 1-mm-thick specimen and its influence on the mechanical properties of the material were investigated by glycerol gas collecting test, static hydrogen charging tensile test, scanning electron microscopy (SEM) test, and microhardness test. The results indicate that the content of diffusible hydrogen tends to be the saturation state when the hydrogen charging time reaches 48 h. The simulation results suggest that the hydrogen concentration distribution can be effectively simulated by ABAQUS and the method can be used to analyze the hydrogen concentration in the material with complex structures or containing multiple microstructures. The influence of hydrogen on the mechanical properties is that the elongation of this material is reduced and the diffusible hydrogen will cause a decrease in the fracture toughness of the material, and thus hydrogen embrittlement (HE) will occur. Moreover, the Young’s modulus E and microhardness are increased due to hydrogen absorption, and the variation value is related to the hydrogen concentration introduced into the specimen.

## 1. Introduction

Hydrogen in nature can exist in the form of hydrogen atoms, hydrogen molecules, hydrogen ions, metallic compounds, hydrocarbons, etc. Hydrogen permeates into metal materials in the form of atoms and is then transmitted in the form of lattice diffusion, stress-induced diffusion, and dislocation migration of hydrogen [1,2,3]. When the hydrogen content in metal materials exceeds a certain threshold, it will cause the loss of material properties and early hydrogen damage will occur. The factors affecting hydrogen damage of metal materials mainly include temperature, hydrogen content, material alloy composition, internal defects, stress and pH value in solution, etc. [4,5,6]. The presence of hydrogen could also increase the concentration of vacancies in the steel, which will more easily form micropores and result in a loss of material properties [7]. When hydrogen diffuses in the metal material, hydrogen will accumulate at the grain boundary of the material. The lattice embrittlement phenomenon will occur when hydrogen gathers at the lattice. If it gathers at the impurities and voids, hydrogen would form high-pressure hydrogen molecules and threat to equipment safety [8,9]. The forms of hydrogen damage in steel mainly include hydrogen embrittlement, hydride embrittlement, high-temperature hydrogen corrosion, hydrogen pressure cracking, etc. Hydrogen embrittlement, including hydrogen-induced plastic loss and hydrogen-induced hysteresis cracking, means that the ductility of metal fracture due to the entry of hydrogen decreases with the decrease of strain rate [10,11], mainly occurring in the temperature range of −100~150 °C, which is the most common form of hydrogen damage. In addition, hydrogen embrittlement behavior is also the main form of hydrogen damage in metal materials studied in the laboratory [12,13,14].

Since the solubility of hydrogen in steel is almost zero at room temperature, the diffusible hydrogen content accounts for more than 80%~90% of the total hydrogen content when hydrogen is introduced into the steel at room temperature [15]. The main factors affecting the content of diffusible hydrogen include temperature, internal defects (including cracks, voids, etc.), hydrogen traps and hydrogen solubility [16,17]. The diffusible hydrogen has a great effect on material properties than dissolved and residual hydrogen, which is an important factor of hydrogen damage to metal materials. For high-pressure hydrogen environments, the content of diffusible hydrogen in steel is also the main part of the total hydrogen content. Therefore, it is particularly important to study the mechanism of hydrogen diffusion, the distribution of hydrogen concentration, and the influence of diffusible hydrogen on material properties, especially when some ab initio methods are available to study the micro-level material properties [18]. Since the 1960s, 2.25Cr-1Mo steel has been widely used for hydrogen service equipment in coal and chemical industries [19], while the traditional 2.25Cr-1Mo steel is still not able to meet the relevant development requirements with respect to the development of equipment towards large size, high parameters, and light weight. After the 1980s, the research on new Cr-Mo steel materials began, and 2.25Cr1Mo0.25V steel has been widely used as the preferred material for equipment for service in high-temperature and high-pressure hydrogen environments due to its having more prominent advantages than other steels. At present, relevant researchers have done some researches on the effects of hydrogen on the mechanical properties of 2.25Cr1Mo0.25V steel. Yang Song et al. [20] investigated the effect of hydrogen on fracture toughness behavior of 2.25Cr-1Mo-0.25V steel and welds and quantitatively assessed fracture toughness by J integral. They suggested the presence of dissolved hydrogen resulted in a significant reduction in fracture toughness and the base metal due to a finer grain size exhibited a superior resistance to HE, and it is proposed that grain size refinement effectively enhances resistance to HE. However, Guerra et al. [21] analyzed hydrogen diffusivity and solubility of 2.25Cr1Mo0.25V steel welded joint and performed related mechanical properties tests, and found that the microstructure of the base metal absorbs more hydrogen than other parts due to martensite and retained austenite, leading to embrittlement of this region. Their work showed that the base metal has lower resistance to HE than other parts of this welding. Although there are some studies on the effect of hydrogen on 2.25Cr-1Mo-0.25V steel, no quantitative relationship has been established between the amount of hydrogen introduced and hydrogen embrittlement degree of the material.

In this study, the analytical solutions of hydrogen concentration distribution in the steel under different hydrogen charging conditions was analyzed based on Fick’s law, and the diffusible hydrogen contents in the specimens were measured by glycerol gas collecting. The effects of diffusible hydrogen on the mechanical properties of 2.25Cr1Mo0.25V steel under different hydrogen charging time conditions were evaluated by static hydrogen charging tensile tests, scanning electron microscopy (SEM) tests, and microhardness tests, respectively. Additionally, the simulation results based on ABAQUS were compared with the analytical solutions to analyze the hydrogen concentration in the materials with complex structures or containing multiple microstructures.

## 2. Experimental Methods and Simulation

### 2.1. Material and Specimens

**Figure 1:**
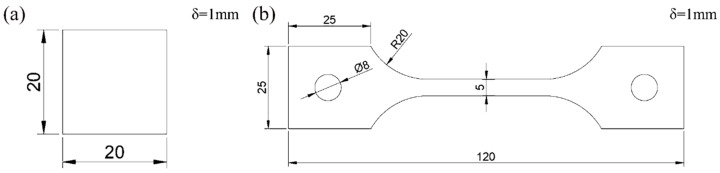
The size of specimens: (**a**) Glycerol gas collecting test sample; (**b**) Tensile test specimen.

The material investigated in this study is 2.25Cr1Mo0.25V ferritic steel provided by Wuyang steel plant (Henan, China). The chemical composition of the steel is shown in Table 1. 2.25Cr1Mo0.25V ferritic steel contains small amounts of vanadium, which is used to increase the mechanical strength of the steel. Samples were cut from the plate, cleaned by ultrasonic cleaner (Skymen, Shenzhen, China). with acetone solution and then dried completely. The sizes of tensile test specimens and diffusible hydrogen content test specimens are shown in Figure 1a,b. The thickness of the two specimens is 1 mm.

Hydrogen charging was performed using the constant current polarization method in electrochemical workstation at room temperature. This method can produce a constant hydrogen concentration on the hydrogen-charging surface of the specimen. Considering that acidic solutions have a large corrosion effect on the specimen, the electrochemical hydrogen charging tests were performed in a sodium hydroxide solution (NaOH solution) at room temperature.

### 2.2. Numerical Analysis of Hydrogen Permeation and Hydrogen Diffusion Process

**Figure 2:**
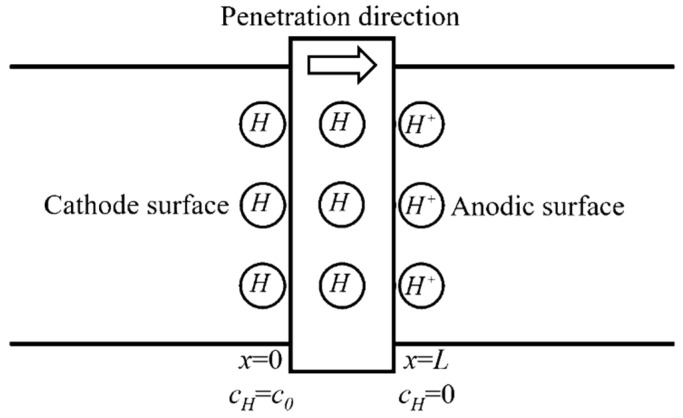
Schematic diagram of the hydrogen permeation model.

The electrochemical hydrogen permeation test (EPT) [22] was applied in this work to measure effective hydrogen diffusivity (*D_eff_*) at room temperature. The double electrolysis cell device (CorrTest, Wuhan, China) consists of using a cell with two separate compartments (as shown in Figure 2). In the cathodic compartment of the cell, a constant cathodic current charge is applied to generate hydrogen ($H^{+}+e\to H$). In the anodic compartment of the cell, a constant anodic potential is applied to oxidize all the hydrogen atoms ($H-e\to H^{+}$) that permeate into the anodic surface entering from the hydrogen-charging surface and produce an oxidation current. The effective hydrogen diffusivity can be calculated from the oxidation current density curve collected by the electrochemical workstation.

According to the theory of electrochemical hydrogen permeation test, the penetration of hydrogen in steel could be regarded as one-dimensional diffusion (diffuse along the thickness of the specimen) and conforms to the second Fick’s Law. Assuming that the hydrogen diffusivity *D* is kept constant (not vary with the increase of hydrogen concentration), the diffusion equation and boundary conditions can be expressed as:

(1) $$\frac{\partial c(x,t)}{\partial t}=D\frac{\partial^{2}c(x,t)}{\partial x^{2}}$$

(2) $$c\left(x=0,t\right)=c_{0};c\left(x=L,t\right)=0;c\left(x,t\right)=0\left(t\leq 0,0\leq x\leq L\right)$$

where *x* is the distance from the hydrogen side, *t* is the hydrogen charging time, *D* is the diffusion coefficient of hydrogen in steel, *c*(*x*, *t*) represents the hydrogen concentration at time *t* at the distance *x* from the hydrogen-charging surface, and $c_{0}$ is the constant concentration of hydrogen on the hydrogen-charging surface of the specimen, depending on the anodic current and thickness of the specimen. According to the hydrogen diffusion equation and boundary conditions, by using the method of Laplace transform, hydrogen concentration profiles at different time *t* could be obtained for the specimen by solving equation of hydrogen diffusion when hydrogen is introduced by method of constant potential polarization.

(3) $$\frac{c(x,t)}{c_{0}}=\sum_{n=0}^{\infty }erfc\left(\frac{2nL+x}{2\sqrt{Dt}}\right)-\sum_{n=0}^{\infty }erfc\left(\frac{2\left(n+1\right)L-x}{2\sqrt{Dt}}\right)$$

where *n* is the natural number, and *L* is the thickness of the specimen. This equation expresses the distribution of the effective hydrogen concentration along the thickness of the specimen during the electrochemical hydrogen permeation test.

**Figure 3:**
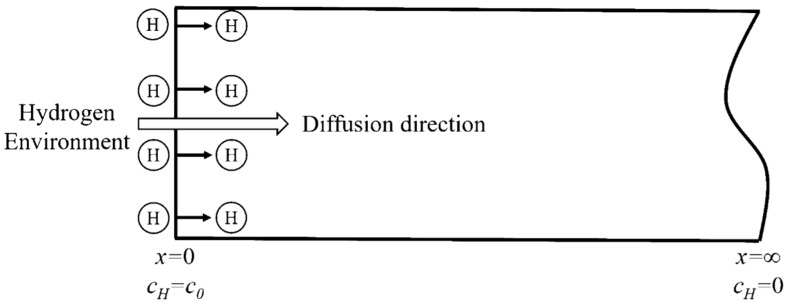
Schematic diagram of the hydrogen diffusion model.

Applying the constant cathodic current in the electrochemical galvanostatic hydrogen charging provides a constant hydrogen concentration on the hydrogen charging surface of the specimen for the hydrogen diffusion into the steel. When adopting the semi-infinite diffusion model, it is assumed that the model is infinite in length (as shown in Figure 3). The diffusion equation conforms the second Fick’s law and the boundary conditions are:

(4) $$c\left(x=0,t\right)=c_{0};c\left(x=\infty ,t\right)=0;c\left(x,t\right)=0\left(t\leq 0,0\leq x\leq L\right)$$

Similarly, the distribution of hydrogen concentration along the thickness direction of the specimen can be obtained by Laplace transform of diffusion equation and boundary conditions. In addition, the change law of hydrogen content *C(H)* with time *t* can be obtained by integrating over all the hydrogen concentrations in the specimen.

(5) $$c\left(x,t\right)=c_{0}\cdot erfc\frac{x}{2\sqrt{Dt}}$$

(6) $$C(H)=\underset{n\to \infty }{\lim}\sum_{i=1}^{n}\frac{c(x_{i},t)\cdot A\Delta x_{i}\cdot d}{L\cdot A\cdot d}=\frac{1}{L}\cdot \int_{0}^{L}c(x,t)dx=\frac{c_{0}}{L}\cdot \int_{0}^{L}erfc\frac{x}{2\sqrt{Dt}}dx$$

where *C(H)* is the total internal hydrogen content absorbed in the galvanostatic hydrogen charging procedure.

### 2.3. Diffusible Hydrogen Content Test

**Figure 4:**
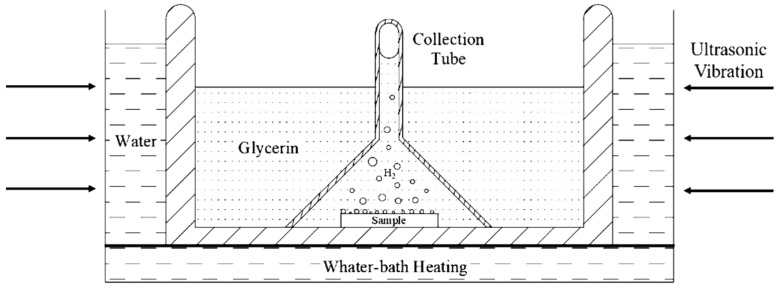
Schematic diagram of glycerin gas collecting and water-bath heating device.

Hydrogen was introduced into specimens electrochemically from 0.2 mol/L NaOH solution in the galvanostatic hydrogen charging. The hydrogen diffusivity measured by EPT is the effective hydrogen diffusivity, which could be understood as the diffusivity of diffusible hydrogen in the steel at room temperature. There are several methods for measuring hydrogen in metallic materials. Thermal desorption spectroscopy (TDS), as a typical method for the measurement of hydrogen, provides some macroscale information about desorption, but this hydrogen originates from all of the possible sites (including the absorbed hydrogen), making it difficult to distinguish the contributions from the different types of hydrogen [23]. Hydrogen embrittlement behavior of the high strength steel is mainly caused by diffusible hydrogen at room temperature, and Yu et al. [24] found that the ductility loss of the material was almost recovered to the original state when the specimens experienced hydrogen charging and then exposed in air for 24 h (i.e., most of the diffusible hydrogen in the specimen overflows). To measure the diffusible hydrogen content in 2.25Cr1Mo0.25V steel after hydrogen charging, glycerol gas collecting is an effective method for measurement of the diffusible hydrogen and all measured hydrogen is diffusible hydrogen. Although glycerol gas collection method is widely applied to measure the gas content in the specimen [25], due to the high viscosity of glycerol and smaller hydrogen bubbles, it is difficult to concentrate the tiny hydrogen bubbles escaping from the specimen to the top of the collection tube. In this work, by reducing the inner diameter of the collection tube and adding ultrasonic vibration method, the tiny hydrogen bubbles keep vibrating under the action of ultrasonic wave and they can gradually concentrate on the top of the funnel. Finally, after 72 h, when all the spilled diffusible hydrogen is concentrated on the top of the collection tube, it can be read out through the test tube at the top. The above operation is repeated under the same conditions, and the experimental results are averaged. The schematic diagram of glycerin gas collection and water bath heating device is shown in Figure 4.

### 2.4. Simulation Analysis of Hydrogen Diffusion in Steel Based on ABAQUS

In this section, Mass Diffusion (MD) module in ABAQUS (2017, Dassault Systèmes Simulia Corp, RI, USA) was used to analyze the distribution of hydrogen concentration in steel after hydrogen was introduced, and its built-in Fick law and chemical potential gradient equation conform to the diffusion law of hydrogen in steel. We compare the results of analytical and numerical solutions of hydrogen diffusion in the specimen and analyze whether the hydrogen concentration distribution can be obtained by simulation. Hydrogen concentration profiles after charging can be estimated by the simulation method when it is difficult to calculate the analytical solutions for the materials with complex structures or containing multiple microstructures.

In this work, we present two simulation models of a simple 2D diffusion problem, which can be compared with the analytical solutions, considering that all hydrogen resides in lattice sites, i.e., that there are no traps in the model. The model of 5 × 1 mm^2^ was established to analyze hydrogen diffusion in the electrochemical hydrogen permeation test process. The model of 5 × 600 mm^2^, to satisfy the condition of semi-infinite source diffusion, was used to analyze hydrogen concentration at constant hydrogen concentration injection. Parameters in the Mass Diffusion module were defined according to material properties, and the diffusivity was the effective hydrogen diffusivity of 2.25Cr1Mo0.25V steel at room temperature.

### 2.5. Mechanical Properties Evaluation

**Figure 5:**
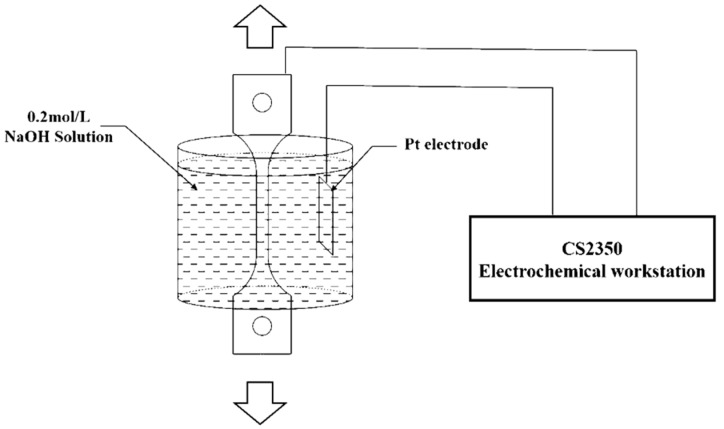
Schematic diagram of static hydrogen charging tensile test device.

Evaluation of mechanical properties was carried out using the static hydrogen charging tensile test at room temperature, and the schematic diagram of the device is shown in Figure 5. The experimental device was fixed on the tensile test machine (model: MJDW-5B, Maijie test equipment, Shandong, China). Firstly, the tested specimens were subjected to electrochemical hydrogen charging for hydrogen absorption. After the specimens had been charged with hydrogen for a period of time, the tensile specimen was immediately stretched to obtain the stress–strain curve of the specimen. Fracture surfaces of the tested tensile specimen were investigated using a scanning electron microscope (model: S3400N, Hitachi, Chiyoda, Japan). In addition, for the hydrogen-charging specimen of 20 × 20 × 1 mm^3^, microhardness tests (device model: VH-1102, Wolpert Wilson, Norwood, CO, USA) were performed along the thickness direction to measure the microhardness of hydrogen-containing specimens.

## 3. Results and Discussion

### 3.1. Hydrogen Permeation Current Data

**Figure 6:**
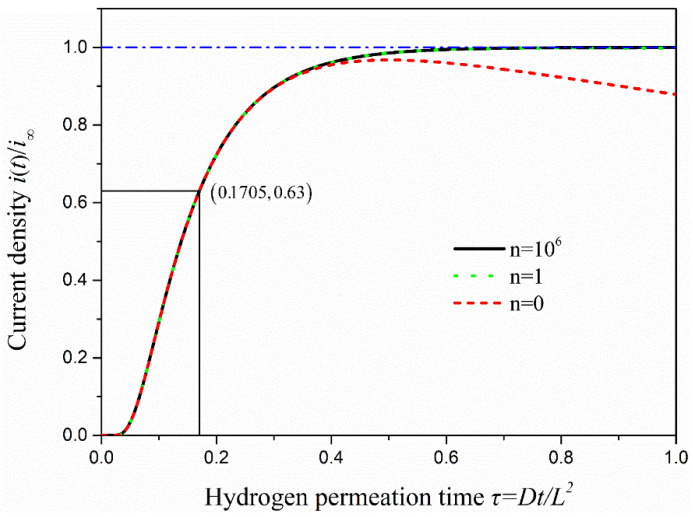
The curve of oxidation current density with hydrogen permeation time under the hydrogen permeation model.

To analyze the hydrogen permeation current data properly, the measured experimental data should be compared with the theoretical model results. The theoretical model used in this study is the constant concentration (CC) model, and its mathematical equation can be expressed as [26]:

(7) $$\frac{i_{t}}{i_{\infty }}=\frac{J_{t}}{J_{\infty }}=\frac{2}{\sqrt{\pi \tau }}\cdot \sum_{n=0}^{\infty }\exp\left[-\frac{{\left(2n+1\right)}^{2}}{4\tau }\right]$$

where $i_{t}$ is the permeation current at instant time *t*, $i_{\infty }$ is the steady state value of the hydrogen permeation current, $J_{t}$ is the hydrogen permeation flux on the anode surface at time t and $J_{\infty }$ is the hydrogen permeation flux under the steady state. The dimensionless time *τ* is equal to *Dt/L*^2^. To describe the change of current density in the process of hydrogen penetration visually, Matlab was used to write the program to draw hydrogen permeation current density curves under *n* = 0, 1 and 10^6^, as is shown in Figure 6.

**Figure 7:**
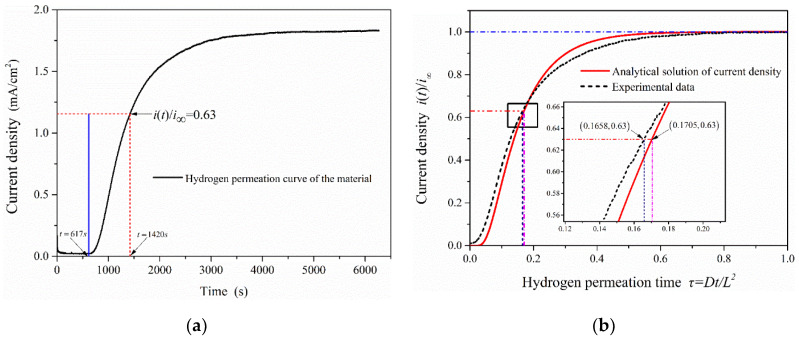
The change curve of oxidation current density in the process of hydrogen permeation: (**a**) Hydrogen permeation curve of 2.25Cr1Mo0.25V steel; (**b**) Comparison of test results with the analytical solutions of oxidation current density obtained under the hydrogen permeation model.

As seen in Figure 6, if *n* takes only 0, the change of oxidation current density is almost the same as when *n* approaches infinity in the early stage of hydrogen permeation. However, there is a trend of decline when permeation time keeps increasing, which is not consistent with the actual situation. When *n* takes 1 or 10^6^, the two curves of hydrogen permeation are almost identical, and the process of hydrogen permeation starts to tend to the steady state when *τ =* 0.4 or so. Therefore, the change of anodic oxidation current density obtained is consistent with the actual situation when the value of *n* is greater than 0 under the CC model. Several methods are commonly used to calculate the effective hydrogen diffusivity (*D_eff_*) under the CC model. In this study, the time-lag method is used to calculate the effective hydrogen diffusivity in steel at room temperature, and it takes *τ =* 1/6 as the calculation value. The effective hydrogen diffusivity (*D_eff_*) in steel can be calculated using the following equation [27].

(8) $$D_{eff}=\frac{\tau L^{2}}{t}=\frac{L^{2}}{6t_{L}}$$

where $t_{L}$ is the lag time (the time from the start of hydrogen charging to *i*(*t*)*/i_∞_* = 0.63) in the electrochemical hydrogen permeation test process. The hydrogen permeation curve of 2.25Cr-1Mo-0.25V steel was measured by EPT (as shown in Figure 7a). It shows that the time to start hydrogen charging is 617 s (hydrogen permeation time *τ =* 0 at this time), and the lag time in the process of hydrogen permeation test is 803 s (*t_L_* = 1420 s − 617 s). The experimental data were compared with the analytical solutions of oxidation current density obtained under the hydrogen permeation model, as is shown in Figure 7b. It can be seen that when the anode oxidation current density reaches *i*(*t*)*/i_∞_* = 0.63, the permeation time under the hydrogen permeation model is *τ =* 0.1705. While the hydrogen permeation time in the experimental data is *τ =* 0.1658, it is almost consistent with the calculated value (*τ =* 1/6) taken in EPT and verifies the validity and accuracy of the test results.

### 3.2. Diffusible Hydrogen Content in the 1-mm-Thick Sample

The specimens were charged with hydrogen applying constant cathodic current in the CS2350 electrochemistry workstation and the charging current was 50 mA/cm^2^. The calculation of hydrogen concentration generated on the charging surface was done according to the following formula [28]: $c_{0}=\alpha \frac{LI_{c}}{DSFd}=\mathrm{const}$. Table 2 shows the relevant data for determining of hydrogen concentration. In this work, the hydrogen concentration on the charging surface is calculated to be about *c_0_* = 2.8 at.ppm. The diffusible hydrogen content measured by glycerol gas collection is the volume (at.mL) of the spilled hydrogen. To indicate the value of the hydrogen content in steel, we used the volume concentration method (that is the volume of hydrogen atoms in a standard state contained in 100 g metal, written as cm^3^/100 g) and 1 ppm = 1.12 cm^3^/100 g after conversion.

**Figure 8:**
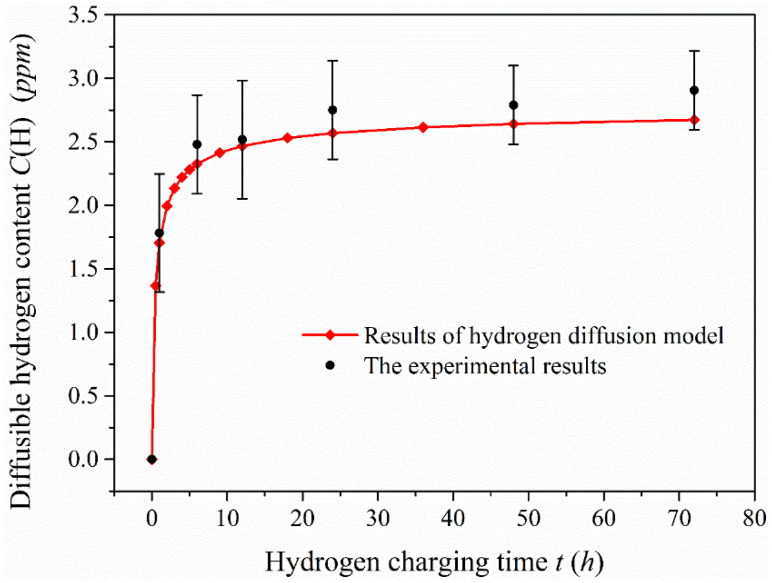
Comparison of diffusible hydrogen content measured in the 1-mm-thick specimen after charging and analytical results under the hydrogen diffusion model.

Figure 8 shows the diffusible hydrogen content in the 1-mm-thick specimen after charging for 6, 12, 24, 48 and 72 h, and the comparison of experimental data with analytical results. Hydrogen absorption rate in the specimen obviously speeds up after charging when the hydrogen charging time is less than 12 h, which indicates that hydrogen diffuses quickly in this material and is sensitive to hydrogen produced. When the hydrogen charging time exceeds a certain value, the diffusible hydrogen content in the 1-mm-thick specimen tends to the saturation state. The change of diffusible hydrogen content tends to be smooth after charging for 24 h, particularly when the hydrogen charging time reaches 48 h. The diffusible hydrogen content is almost consistent with that measured after charging for 72 h, which implies that the diffusible hydrogen content in the specimen is close to the saturation value. The saturation value of diffusible hydrogen content is equal to hydrogen concentration on the charging surface. According to the comparison of the experimental data and the analytical results under the hydrogen diffusion model, the results of both are very close. This also indicates that the diffusible hydrogen content obtained by this method could be used as a parameter to evaluate hydrogen embrittlement behavior.

### 3.3. Simulation Results and Analysis

#### 3.3.1. Simulation Results of the Hydrogen Permeation Model

**Figure 9:**
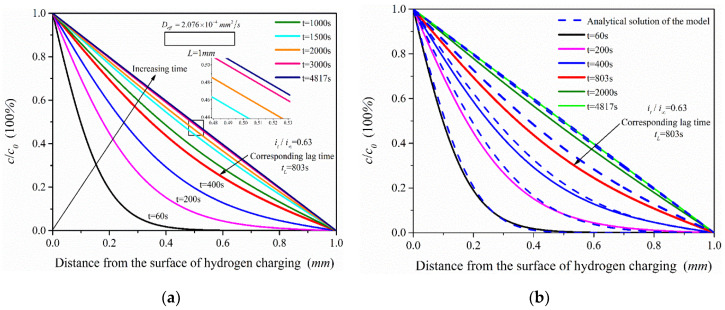
Hydrogen concentration distribution under different permeation time: (**a**) Finite element simulation results of the hydrogen permeation model; (**b**) Comparison of the analytical solution of the hydrogen permeation model and the simulation results.

**Figure 10:**
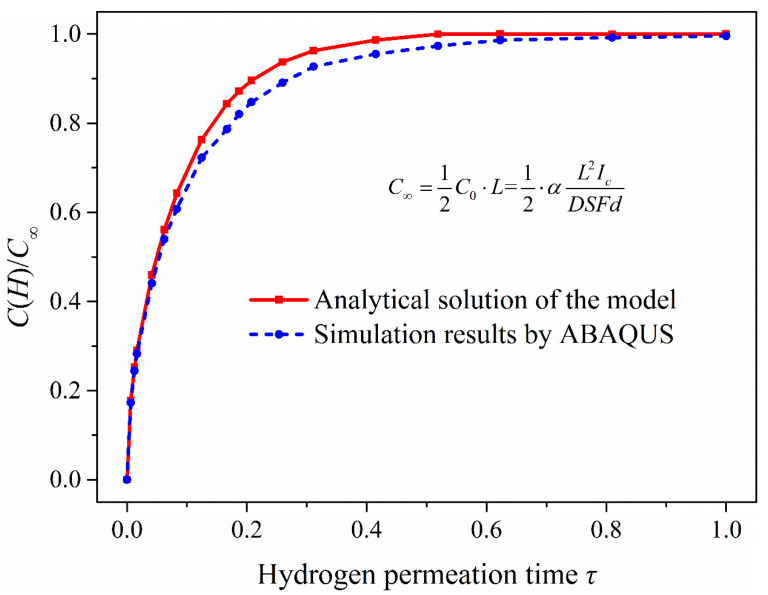
The change trend of hydrogen content in the process of hydrogen permeation.

The effective hydrogen diffusivity (*D_eff_*) of 2.25Cr1Mo0.25V steel was obtained as 2.076 × 10^−4^ mm^2^/s by electrochemical hydrogen permeation test. The hydrogen permeation time was 4817 s when *τ = Dt/L*^2^ = 1. The simulation analysis time was set as 60, 200, 400, 803, 2000, 3000 and 4817 s, and the simulation results of ABAQUS were compared with the analytical solutions (see Equation (3)) of the hydrogen permeation model, as shown in Figure 9. The change trend of hydrogen content in the hydrogen permeation model with time is shown in Figure 10.

According to the simulation results (Figure 9a), the hydrogen concentration in the hydrogen permeation model increases with permeation time and tends to the steady state. In addition, it shows that when *t =* 1500 s (*τ =* 0.4152), the hydrogen permeation process is close to the steady state; especially when the permeation time is 2000 s and 3000 s, the distribution of hydrogen concentration is very close to the steady state (when *t =* 4817 s (*τ =* 1)). The hydrogen concentration in the hydrogen permeation model is linearly distributed when hydrogen permeation process reaches the steady state. Comparing the analytical solutions of the model, the hydrogen concentration distribution and the change trend of hydrogen content (Figure 10) of the two are almost consistent, with only slight deviation in the middle process of hydrogen permeation. Considering hydrogen diffusion also driven by the gradient of chemical potential in ABAQUS mass diffusion module, the simulation results are slightly different from the analytical solutions of the model obtained according to Fick law. With the increasing hydrogen content in the model, chemical potential gradient equation would have a certain inhibitory effect on hydrogen diffusion compared with the diffusion based on Fick law alone, especially in the middle state of hydrogen permeation process, which leads to the simulation results slightly lower than the analytical solutions of the hydrogen permeation model. Figure 10 shows that the simulated hydrogen content is slightly lower than the analytical solutions of the model only in the middle of hydrogen permeation, but the overall change trend of hydrogen content in the hydrogen permeation model is very close.

#### 3.3.2. Simulation Results of the Hydrogen Diffusion Model

**Figure 11:**
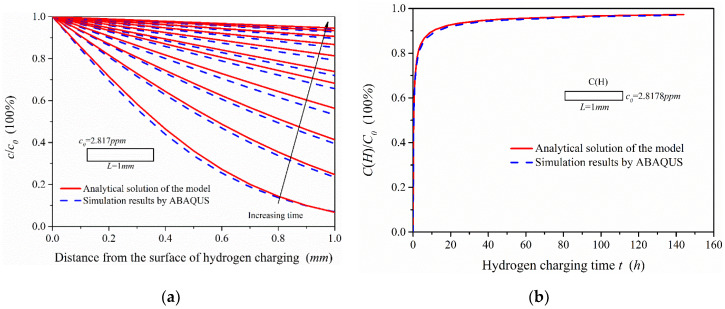
Comparison of simulation results and analytical solutions of the model: (**a**) Hydrogen concentration distribution in the 1-mm-thick sample under different hydrogen charging time; (**b**) the change trend of hydrogen content in the 1-mm-thick model with hydrogen charging time.

To combine the experimental study, the part of the model with a thickness of 1 mm from the surface of hydrogen charging was selected to analyze the hydrogen concentration distribution in the 1-mm-thick model. In addition, the hydrogen charging time was 0.2, 0.5, 1, 2, 4, 6, 12, 24, 48, 96 and 144 h. The simulation results were compared with the analytical solutions (see Equation (5)) of the model and the change trend of hydrogen content in the 1-mm-thick sample with time is shown in Figure 11.

Figure 11a shows that the ABAQUS simulation results were slightly less than the analytical solutions of the hydrogen diffusion model. This is because the analytical solutions are obtained in the condition of semi-infinite source diffusion, and the hydrogen activity at infinity is zero. Since the infinite length of the model cannot be realized in simulation, the hydrogen activity on the other side of the model (at *x =* 600 mm) was set to zero, which inhibits the hydrogen diffusion ability in the simulation. If the length of hydrogen diffusion simulation model is continuously increased, the simulation results can be closer to the analytical solutions of the model. It can be seen from Figure 11b, under the condition of electrochemical hydrogen charging, that the change of hydrogen content in the 1-mm-thick model is obvious in the early stage of hydrogen charging, which is related to the effective hydrogen diffusivity in steel. When the hydrogen charging time reaches 48 h, the hydrogen content is close to the steady state. According to the comparison of the analytical solutions and the simulation results, the two change curves of hydrogen content are almost the same. There is, however, a slight difference in the hydrogen concentration distribution, which has little influence on the hydrogen content in the 1-mm-thick sample.

In conclusion, the hydrogen diffusion process can be effectively simulated by ABAQUS and this method can also be used to obtain the hydrogen concentration distribution and the change of hydrogen content in the materials with complex structures or containing multiple microstructures.

### 3.4. Effects of Electrochemical Hydrogen Charging on the Mechanical Properties

**Figure 12:**
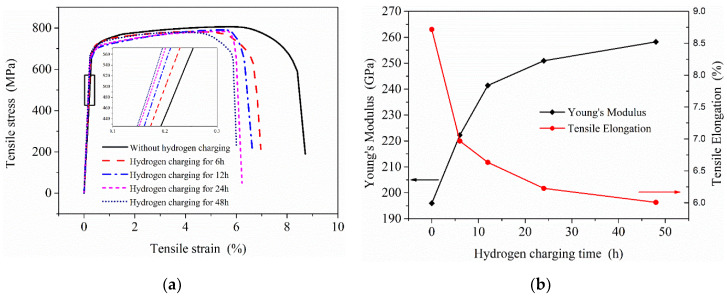
Effects of hydrogen charging on the mechanical properties of 2.25Cr1Mo0.25V steel: (**a**) stress–strain curves of samples under different hydrogen charging time; (**b**) the change of Young’s modulus and tensile elongation.

Through the static hydrogen charging tensile test device, tensile tests were performed on tensile specimens after charging for 0, 6, 12, 24 and 48 h. The stress–strain curves of 2.25Cr1Mo0.25V steel specimens after charging are presented in Figure 12.

The effects of introducing hydrogen on the mechanical properties of 2.25Cr1Mo0.25V steel mainly include Young’s modulus, elongation and tensile strength (Figure 12a). The elongation (total elongation of the tensile specimen) and Young’s modulus of this material were significantly affected by hydrogen, while the yield and tensile strength did not change notably on hydrogen charging. It can be seen from the experimental results that the elongation of the material and the reduction rate in elongation are decreasing with charging time, which is very similar to the change trend of hydrogen content. Additionally, the Young’s modulus of this material shows a gradual increase trend that is similar to the increase trend of concentration yet, indicating that atomic bonding force and dislocation configuration in the specimen might change after hydrogen charging to cause the variation of Young’s modulus E. Clearly, electronic effects and the spatial changes must be considered to explain the variation of E with hydrogen concentration. This is coincident with Wriedt’s findings, and their work indicates that every 1% (atomic fraction) hydrogen will increase the Young’s modulus E of vanadium, niobium and tantalum [29]. Recently, Yi-sheng Chen et al. used cryo-transfer atom probe tomography to observe hydrogen at an incoherent interface, which can act as trapping sites between niobium carbides and the surrounding steel [30]. With our present state of knowledge, no single mechanism can completely explain the variation of Young’s modulus E observed in this V-containing material by hydrogen and it is necessary to further study the influence mechanism of hydrogen on the materials with different microstructures at the microscopic level.

**Figure 13:**
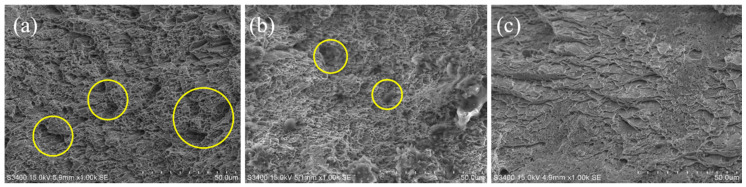
SEM images of tensile specimens under different hydrogen charging time: (**a**) Without hydrogen charging; (**b**) Hydrogen charging for 24 h; (**c**) Hydrogen charging for 48 h.

To understand the fracture mechanisms of this material after hydrogen charging, the fracture morphology of specimens was characterized by scanning electron microscope (SEM), as shown in Figure 13. As a result, a large number of voids and dimples were found on the fracture surface of specimen with hydrogen-free (Figure 13a). The voids and dimples are decreasing in the SEM images of the specimens as a result of hydrogen absorption, and this is related to the charging time (hydrogen content). This result indicates that the fracture toughness of the material decreases significantly, which is consistent with the tensile test results. Therefore, without considering the generation of cracks, hydrogen embrittlement (HE) may be the main form of hydrogen damage caused by hydrogen introduced at room temperature.

**Figure 14:**
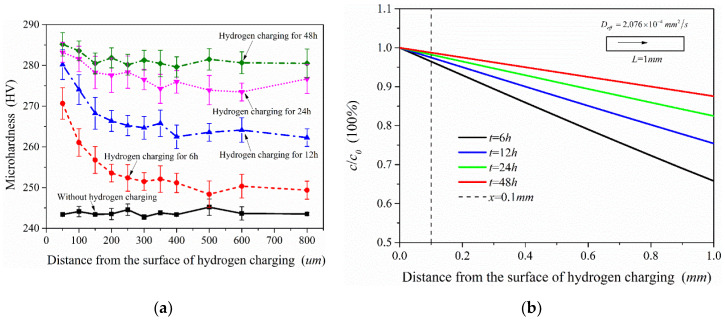
Microhardness test results of the samples under different hydrogen charging time (**a**) and diffusible hydrogen concentration distribution after charging (**b**).

Figure 14a shows the microhardness test results of the samples after different charging time. For the sample without hydrogen charging, the microhardness of the material is close along the thickness direction. In contrast to the hydrogen-free case, the microhardness of the samples after hydrogen charging presents an increasing trend, but the increased value was not uniform, which is related to the hydrogen concentration at different locations. According to the test results, the microhardness showed a decreasing trend along the thickness direction (hydrogen diffusion direction) when the hydrogen charging time was only 6 h. With the increase of hydrogen charging time, the overall microhardness of the sample also increased. This shows that the microhardness of this material was extremely sensitive to the hydrogen introduced, and the higher hydrogen concentration in the sample, the greater its microhardness. Figure 14b shows the diffusible hydrogen concentration distribution after charging according to Equation (5). It is acceptable that the microhardness distribution measured in the experiment is very similar to the hydrogen concentration distribution under the hydrogen diffusion model. Therefore, the distribution of hydrogen concentration in the material could also be shown by microhardness test results.

## 4. Conclusions

In this study, analytic equations were derived on the basis of mathematical models based on Fick law during the electrochemical hydrogen permeation test and galvanostatic hydrogen charging. Galvanostatic hydrogen charging was performed in 2.25Cr-1Mo-0.25V steel to introduce hydrogen at room temperature, and the results were mainly based on glycerin gas collecting tests, simulation and mechanical properties evaluation after charging. The main conclusions are as follows.

(1) The diffusible hydrogen content in the 1-mm-thick sample is very close to the analytical solutions under different hydrogen charging time, and it is close to the saturation value when the hydrogen charging time reaches 48 h.
(2) The simulation results and the analytical solutions under the hydrogen permeation model are close, only slightly lower than the analytical solutions in the middle of hydrogen permeation. Comparing the analytical solutions of hydrogen diffusion model, the simulation results are nearly identical. Therefore, the simulation method based on MD module in ABAQUS can be used to analyze the hydrogen concentration distribution in the material with complex structures or containing multiple microstructures.
(3) Hydrogen introduced will decrease the elongation of this material and increase its Young’s modulus, and it has no notable influence on yield strength and tensile strength. The reduction of the dimples in the SEM images of the tensile specimens indicates that hydrogen charging will cause a degradation in the fracture toughness of the material and hydrogen embrittlement will occur.
(4) Hydrogen absorption will also increase the microhardness of this material, and this is related to the hydrogen concentration introduced. Additionally, the microhardness tests of the material may be used to analyze the hydrogen concentration distribution inside the material as well.

## Figures and Tables

**Table 1:** Chemical composition of test specimen (wt %).

Element	C	S	P	Si	Mn	Cr	Ni	Mo	V
content	0.14	0.006	0.005	0.06	0.60	2.31	0.08	1.07	0.35

**Table 2:** Data for determining of hydrogen concentration on the charging surface.

Steel Grade	Density *d* (g/cm^3^)	*I* (mA)	*S *(cm^2^)	*D_eff_* (m^2^/s)
2.25Cr1Mo0.25V	7.20	200	4	2.076 × 10^−4^
